# Penicillin allergy de‐labelling by non‐allergy specialists in the elective surgical setting: A mixed‐methods systematic review protocol

**DOI:** 10.1002/anr3.70072

**Published:** 2026-06-21

**Authors:** A. Littlejohns, A. Al‐Hakim, L. Savic, A. Kirby, H. Pandit, N. Rousseau

**Affiliations:** ^1^ Leeds NIHR Biomedical Research Centre Leeds UK; ^2^ Department of Theatres and Anaesthesia Leeds Teaching Hospitals NHS Trust Leeds UK; ^3^ School of Medicine University of Leeds Leeds UK; ^4^ Department of Clinical Immunology and Allergy Leeds Teaching Hospitals NHS Trust Leeds UK; ^5^ Department of Microbiology Leeds Teaching Hospitals NHS Trust Leeds UK; ^6^ Department of Orthopaedic Surgery Leeds Teaching Hospitals NHS Trust Leeds UK

**Keywords:** de‐labelling, elective surgery, non‐allergist, penicillin allergy, peri‐operative care

## Abstract

Penicillin allergy labels are common in elective surgical patients, although most are incorrect. These labels often prevent the use of first‐line surgical prophylaxis, and alternative antibiotic choices are associated with patient harm and antimicrobial resistance. There is a need for effective pre‐operative penicillin allergy de‐labelling services, but demand cannot be matched by allergists alone, making non‐allergist‐led approaches particularly important. Such services have been implemented in a range of health care settings, although their design, level of allergist supervision, implementation and clinical effectiveness vary. The elective surgical population is a promising cohort for these interventions, but has been studied less than inpatient groups. We aim to synthesise quantitative and qualitative evidence on non‐allergist‐led penicillin allergy de‐labelling interventions in elective surgical populations. Studies involving penicillin allergy de‐labelling by non‐allergist healthcare professionals in the elective surgical setting will be eligible, including quantitative studies of clinical effectiveness and economic evaluations, and qualitative studies exploring experiences, perceptions or implementation factors. A mixed‐methods systematic review will be conducted using a convergent segregated approach in line with Joanna Briggs Institute methodology. Systematic searches will be performed across MEDLINE, Embase, CINAHL, Web of Science, Scopus and ClinicalTrials.gov. Studies will be screened and data extracted independently by two reviewers. Quantitative and qualitative findings will be synthesised separately and then integrated to identify convergence, divergence and complementarity.

## Introduction

First‐line surgical prophylaxis commonly involves β‐lactam antibiotics [1]. However, an estimated 10% of surgical patients carry a penicillin allergy label and, as a result, are often denied penicillin or other β‐lactam antibiotics peri‐operatively [2]. Penicillin allergy labels have been reported to increase the odds of surgical site infections by 50%, which is attributable to inferior alternative antibiotics [3]. The use of non‐β‐lactam antibiotics as prophylaxis has been shown to increase the odds of surgical site infection by 80% [4]. This increased risk is seen across all types of surgical site infection, all categories of surgical procedures and all non‐β‐lactam agents [4]. The use of alternative antibiotics also increases the risk of healthcare‐associated infections and drug‐resistant infections, such as *Clostridioides difficile* colitis, vancomycin‐resistant enterococcus and methicillin‐resistant *Staphylococcus aureus* infections [5, 6, 7]. Alternative antibiotics often have a broader spectrum, which is a driver for antimicrobial resistance [8, 9, 10]. Further harms include an increased risk of anaphylaxis when alternative agents with a higher incidence of hypersensitivity reactions, such as teicoplanin, are used [11].

On testing, over 95% of penicillin allergy labels are false, meaning that this increased peri‐operative harm is avoidable [5, 6, 12, 13]. Surgical waiting lists, or surgical preparedness pathways, present an opportunity to investigate penicillin allergy labels ahead of elective surgery [14]. The investigation and removal of labels, where appropriate, is termed ‘de‐labelling’. De‐labelling interventions ahead of surgery could improve peri‐operative care for individual patients and improve peri‐operative antimicrobial stewardship [3].

Given the scale of demand for penicillin allergy de‐labelling, specialist allergy services are unlikely to have sufficient capacity to provide this service across the UK [15]. Under guidance from the UK National Institute for Health and Care Excellence, only a small proportion of patients with penicillin allergy labels are suitable for referral to these specialist services [16]. Therefore, non‐allergists are required to undertake such allergy investigations [15]. National guidelines support the set‐up of non‐allergist‐led de‐labelling services in the UK [15]. There has been substantial clinical activity in this field in primary and secondary care, with many centres across the UK developing non‐allergist‐led de‐labelling services [17]. However, there is significant heterogeneity in how these services are provided and varying levels of supervision from allergy specialists. There also appear to be issues with implementation, and penicillin allergy de‐labelling by non‐allergists is increasingly recognised as a complex intervention [18, 19].

Elective surgical patients are a promising cohort for de‐labelling interventions [20, 21]. These patients are already engaged with the healthcare system and are very likely to require antibiotics, such as penicillin or other β‐lactam antibiotics, in their peri‐operative period. They are also more medically stable than other cohorts, such as inpatients on medical or critical care wards. However, potential obstacles include knowledge gaps amongst anaesthetists regarding allergy investigations and a lack of confidence in de‐labelling processes amongst anaesthetists [2, 14, 22]. De‐labelling in the pre‐operative setting has been less extensively studied than in other secondary care or primary care settings.

Existing reviews have examined penicillin allergy de‐labelling [23, 24, 25, 26]. However, these syntheses largely include heterogeneous populations and settings, limiting context‐specific conclusions for pre‐operative elective surgical pathways. Previous reviews also include studies which are either allergist‐led or involve substantial allergist input, making it difficult to draw conclusions about non‐allergist‐led services. Prior reviews have predominantly focused on quantitative effectiveness outcomes, with limited integration of implementation evidence, such as feasibility, acceptability and barriers to delivery. This is particularly important as penicillin allergy de‐labelling by non‐allergists is complex and requires changes in clinical behaviour, training and organisational processes. Recent primary research has demonstrated the feasibility of non‐allergist‐led de‐labelling and highlighted key implementation challenges within healthcare systems. These findings underscore the need for a focused synthesis of evidence specific to non‐allergist‐led models in elective surgical care pathways.

We therefore aim to synthesise evidence on non‐allergist‐led penicillin allergy de‐labelling interventions in elective surgical patients, including their effectiveness, safety, economic impact and implementation. Specifically, this review will examine what approaches have been reported; what is known about the experiences, perceptions and attitudes of patients and healthcare professionals; what barriers and enablers influence implementation; and how quantitative outcomes and qualitative insights complement or contrast with one another. To our knowledge, this specific combination of setting, delivery model and mixed‐methods methodology has not been previously synthesised.

## Methods

This systematic review will be conducted in accordance with the Joanna Briggs Institute methodology for mixed‐methods systematic reviews [27]. This approach supports the systematic identification, appraisal and synthesis of both quantitative and qualitative evidence, followed by integration of the findings to provide a comprehensive understanding of the review question.

Study selection, methodological appraisal, data extraction and data synthesis will be performed by two independent reviewers. Disagreements will be resolved through discussion or, where consensus cannot be reached, by consultation with a third reviewer.

### Eligibility criteria

The review will include studies of patients aged 16 years or older with a penicillin allergy label who are on the waiting list for elective surgery, regardless of surgical specialty. The allergy label may be documented in primary or secondary care notes or reported by the patient. Where studies included both adult and paediatric populations, adult data will be included, if they can be extracted separately. Where studies include multiple settings, only data relating to pre‐operative patients will be included, if they can be extracted separately. Studies from all countries will be eligible. Primary and secondary care settings will be included, although most studies are expected to be conducted in secondary care.

The intervention of interest is investigation of penicillin allergy labels and subsequent removal or confirmation of labels as appropriate. This may include skin testing, skin testing followed by challenge testing, direct challenge testing or clinical de‐labelling. Interventions designed to support penicillin allergy investigations, such as training for non‐allergists and development of de‐labelling protocols, will also be included. The intervention must be delivered by non‐allergy specialists and may be performed by any healthcare professional other than a clinical immunologist or allergist. Interventions which include allergists within the pathway, for example through advisory input, will be included provided that most of the intervention is delivered by non‐specialists. Details of allergist involvement and supervision will be recorded.

Eligible interventions must take place in the pre‐operative elective surgical setting amongst patients listed for and awaiting elective surgery. Studies conducted in inpatient surgical wards with acute surgical patients or postoperative surgical patients will be excluded.

Studies with and without comparators will be included. Where comparators are reported, all types will be eligible, including usual care and allergist‐led de‐labelling pathways.

For the quantitative component, the primary outcomes will be successful de‐labelling rate and adverse drug reactions during testing. Secondary outcomes may include persistence of de‐labelling at three or six months; peri‐operative use of β‐lactam antibiotics; use of β‐lactam antibiotics in the subsequent year; clinical outcomes, such as surgical site infection, postoperative complications and length of stay; long‐term antibiotic use; uptake and participation rates; economic outcomes, including cost‐effectiveness, cost‐utility, cost–benefit and cost‐minimisation; and implementation‐related quantitative measures, such as acceptability.

For the qualitative component, studies examining implementation and feasibility will be eligible. This includes stakeholder experiences and perceptions; acceptability and feasibility of the intervention; barriers and facilitators to implementation; and implementation fidelity, including adherence to the intended intervention design, extent of intervention coverage, quality of delivery and participant responsiveness.

Quantitative, qualitative and mixed‐methods studies will be included. Quantitative studies may be prospective or retrospective, observational or interventional. Qualitative studies may use narrative or phenomenological approaches, including focus groups, interviews and observation. Mixed‐method studies will be included if the quantitative or qualitative components can be clearly extracted, including studies in which data have been qualitised or quantitised. Only studies published in English will be included.

### Information sources and search strategy

A preliminary search of MEDLINE was undertaken to identify relevant terms used in titles and abstracts, and these informed the full search strategy. The MEDLINE search strategy is provided in Supporting Information Appendix S1 and will be adapted for use in each database. The following electronic databases will be searched from inception to the date of search: Embase (via Ovid), MEDLINE (via Ovid), Scopus (via Elsevier), CINAHL (via EBSCOhost), Web of Science Core Collection (via Clarivate) and the Cochrane Library. ClinicalTrials.gov will be searched to identify ongoing or unpublished studies. Additional studies will be identified through backward and forward citation searching, conference proceedings and contact with relevant authors.

### Selection process

All identified records will be uploaded into Covidence (Veritas Health Innovation Ltd., Melbourne, Australia) and duplicates will be removed. Titles and abstracts will be screened independently by two reviewers against our inclusion criteria. Potentially relevant studies will undergo full‐text review. Reasons for exclusion at the full‐text stage will be recorded. The study selection process will be reported in full and presented in a Preferred Reporting Items for Systematic Reviews and Meta‐analyses (PRISMA) flow diagram [28].

### Assessment of methodological quality

Eligible studies will be critically appraised independently by two reviewers using the appropriate Joanna Briggs Institute critical appraisal tools according to study design [29]. The results of the critical appraisal will be reported in narrative and tabular form. All included studies, regardless of methodological quality, will proceed to data extraction and synthesis. Appraisal findings will be used to inform the interpretation of the results and the strength of the conclusions.

### Data extraction

For quantitative studies, including the quantitative component of mixed‐methods studies, the following data items will be extracted where available: study characteristics, including authors, year of publication, country, study design, setting, surgical speciality and sample size; participant characteristics, including age, sex, comorbidities, description of the penicillin allergy label and risk stratification; and intervention characteristics informed by the Template for Intervention Description and Replication (TIDieR) checklist [30], including type of de‐labelling approach (direct oral challenge, skin testing and clinical history‐based de‐labelling), healthcare professional delivering the intervention, level of allergist involvement or supervision, setting and timing of intervention and components of the intervention (risk stratification tools, protocols and training). Comparator details, outcome measures and results, and economic data will also be extracted. Quantitative outcome data will include de‐labelling outcomes (proportion assessed and proportion de‐labelled), antibiotic use, safety outcomes (adverse drug reactions and anaphylaxis), clinical outcomes (surgical site infections, complications and length of stay), longer‐term outcomes (antibiotic use postoperatively and persistence of de‐labelling), implementation‐related measures and economic outcomes, such as type of analysis, analytic perspective, time horizon, cost components and outcome measures (Quality‐Adjusted Life Years).

For qualitative studies, including the qualitative component of mixed‐methods studies, extracted data will include study characteristics, participant characteristics, methodological details, context of the intervention, findings and implementation factors. This will include participant type, data collection methods, analytical approach, description of the de‐labelling pathway and healthcare setting, reported themes or categories, supporting quotation, authors' interpretations and reported barriers and facilitators, acceptability, feasibility and perceptions of safety or effectiveness. Where necessary, authors will be contacted for missing or clarifying information.

### Data synthesis and integration

A convergent segregated synthesis approach will be used [27]. Quantitative and qualitative evidence will first be synthesised separately and then integrated.

Quantitative findings will initially be summarised in tabular and narrative form. Where studies are sufficiently homogeneous, meta‐analysis will be undertaken. Clinical and methodological heterogeneity will be assessed based on study characteristics, intervention characteristics and outcomes. Statistical heterogeneity will be assessed using the chi‐squared test and quantified using the I^2^ statistic, with interpretation undertaken cautiously, particularly where the number of studies is small. Where meta‐analysis is appropriate, a random‐effects model will be used to account for variation in intervention delivery and implementation context across studies. Between‐study variance will be estimated using tau‐squared. Dichotomous outcomes will be summarised as risk ratios or odds ratios with 95% confidence intervals. Continuous outcomes will be summarised using mean differences or standardised mean differences, as appropriate. If at least 10 studies are included in a meta‐analysis, publication bias will be assessed using funnel plots and statistical tests such as Egger's test. Where sufficient data are available, subgroup analyses may be conducted according to type of de‐labelling intervention. Where meta‐analysis is not appropriate, a structured narrative synthesis will be conducted, grouping studies by intervention type and outcome and summarising patterns across studies.

Qualitative data will be synthesised using a thematic synthesis. Extracted findings, including themes, categories and supporting quotations, will be coded inductively. Codes will then be organised into descriptive themes and interpreted further to generate higher‐order analytical themes. Where appropriate, findings will be mapped to relevant implementation constructs, such as barriers, facilitators, acceptability and feasibility, to support interpretation. A clear audit trail will be maintained to link original study findings to synthesised themes. Where qualitative data are limited, findings will be presented in a structured narrative format that preserves the context and meaning of individual studies.

The two strands of evidence will then be integrated by juxtaposing quantitative and qualitative findings to identify convergence, divergence and complementarity. Quantitative outcomes, such as effectiveness and safety, will be compared with qualitative findings, such as perceptions of safety and acceptability. Qualitative evidence will be used, where relevant, to help explain variation in quantitative findings, such as differences in implementation or uptake, and to identify areas where one type of evidence is lacking. Integrated findings will be presented narratively and, where appropriate, supported by joint displays or tables linking quantitative and qualitative evidence explicitly.

### Certainty of evidence

Established certainty frameworks are not routinely applicable to mixed‐methods systematic reviews integrating qualitative and quantitative evidence. Therefore, a formal certainty assessment will not be undertaken, consistent with Joanna Briggs Institute guidance for mixed‐methods systematic reviews [27].

## Supporting information


**Appendix S1.** MEDLINE search strategy.
