# Supplementary material for: Penicillin allergy de‐labelling by non‐allergy specialists in the elective surgical setting: A mixed‐methods systematic review protocol
Source: Anaesth Rep. 2026 Jun 21;14(1):e70072. doi: 10.1002/anr3.70072 (PMC13283782; doi:10.1002/anr3.70072)
Supplement: Supplementary file 1 — Appendix S1. MEDLINE search strategy. [file ANR3-14-e70072-s001.docx]

**Supporting Information**

**Appendix S1. MEDLINE search strategy.**

(
"Drug Hypersensitivity"[Mesh]
OR "Penicillins/adverse effects"[Mesh]
OR "beta-Lactams/adverse effects"[Mesh]
OR "penicillin allergy"[tiab]
OR "beta-lactam allergy"[tiab]
OR "penicillin hypersensitiv*"[tiab]
)

AND

(
delabel*[tiab]
OR "de-label*"[tiab]
OR "allergy delabelling"[tiab]
OR "risk stratification"[tiab]
OR "direct oral challenge"[tiab]
OR "skin testing"[tiab]
)

AND

(
nurse*[tiab]
OR pharmacist*[tiab]
OR surgeon*[tiab]
OR anaesthetist*[tiab]
OR anesthetist*[tiab]
OR anesthesiologist*[tiab]
OR "non-allergist*"[tiab]
OR "non specialist*"[tiab]
)

AND

(
"Surgical Procedures, Operative"[Mesh]
OR "Perioperative Care"[Mesh]
OR surg*[tiab]
OR perioperativ*[tiab]
OR preoperativ*[tiab]
OR preassessment[tiab]
OR "pre-admission"[tiab]
OR "preoperative clinic"[tiab]
OR anaesthe*[tiab]
OR anesthe*[tiab]
)
